# Molecular Characterization of Klebsiella pneumoniae Clinical Isolates Through Whole-Genome Sequencing: A Comprehensive Analysis of Serotypes, Sequence Types, and Antimicrobial and Virulence Genes

**DOI:** 10.7759/cureus.58449

**Published:** 2024-04-17

**Authors:** Vinay Kumar Moses, Venkataramana Kandi, Vallab Ganesh Bharadwaj, Tarun Kumar Suvvari, Eswar Podaralla

**Affiliations:** 1 Microbiology, Government Medical College, Karimnagar, Karimnagar, IND; 2 Clinical Microbiology, Prathima Institute of Medical Sciences, Karimnagar, IND; 3 Microbiology, Trichy Sri Ramasamy Memorial Medical College Hospital & Research Centre, Tiruchirapalli, IND; 4 General Medicine, Rangaraya Medical College, Kakinada, IND; 5 Research, Squad Medicine and Research, Visakhapatnam, IND; 6 Internal Medicine, Temple University, Philadelphia, USA

**Keywords:** multidrug resistant (mdr), whole-genome sequencing (wgs), hypervirulent k. pneumoniae (hvkp), antibiotic resistance, virulence genes, multilocus sequence typing (mlst), next-generation sequencing (ngs), klebsiella pneumoniae, molecular methods, antimicrobial resistance (amr)

## Abstract

Introduction

Antimicrobial resistance (AMR) has become a menace, spreading among bacterial species globally. AMR is now recognized as a silent pandemic responsible for treatment failures. Therefore, an effective surveillance mechanism is warranted to understand the bacterial species isolated from human clinical specimens. The present study employed next-generation sequencing (NGS) or whole-genome sequencing (WGS) to identify the resistance and virulence genes, sequence type, and serotypes.

Methods

This study included 18 multidrug-resistant (MDR) *Klebsiella pneumoniae* (*K*. *pneumoniae*) isolates obtained from patients suffering from different infections attending the Prathima Institute of Medical Sciences, Karimnagar, India. All isolates were identified, and antimicrobial susceptibility profiles were determined through conventional microbiological techniques and confirmed by automated systems. All the isolates were investigated using NGS or WGS to identify the genes coding for resistance, such as extended-spectrum beta-lactamases (ESBLs), metallo-beta-lactamases, and virulence genes. Multilocus sequence typing (MLST) was conducted to identify the sequence types, and Kleborate analysis was performed to confirm the species, genes for AMR, and virulence and evaluate the capsular polysaccharide (KL) and cell wall/lipopolysaccharide (O) serotypes carried by the isolates.

Results

The mean age of the patients was 46.11±20.35 years. Among the patients included, 12 (66.66%) were males and 6 (33.33%) were females. A high percentage (>50%) of hypervirulent *K*. *pneumoniae *(hvKp) strains that had genes coding for AMR and plasmids having the potential to carry *bla*_NDM_ and resistance genes were observed. Among the isolates, 16 (88.88%) revealed the presence of multiple antibiotic-resistant genes with evidence of at least one gene coding for beta-lactamase resistance. There was a high prevalence of *bla*_SHV_ (17/18; 94.44%) and *bla*_CTX-M-15_ (16/18; 88.88%) AMR genes. Other AMR genes identified included *bla*_TEM_ (83.33%; 15/18) and *bla*_OXA_ (14/18; 77.77%). Two (11.11%) strains each showed the presence of *bla*_NDM-1_ and *bla*_NDM-5_ genes. The virulence genes identified included *gapA*, *infB*, *mdh*, *pgi*, *phoE*, *rpoB*, *tonB*, and *ybt*. The most frequent *K*. *pneumoniae* serotypes found were KL51:O1v2 (3/18, 16.66%), KL17:O1v1 (3/18, 16.66%), and KL64:O2v1 (3/18, 16.66%). KL64 (4/18; 22.22%) was the most common capsular serotype identified among the isolates. The most frequent MLST-based sequence type (ST) identified included ST-147 (5/18, 27.77%), followed by ST-231 (3/18, 16.66%) and ST-101 (2/18, 11.11%).

Conclusions

The molecular analysis of *K*. *pneumoniae* isolates revealed multiple AMR, plasmid, and virulence genes. Additionally, many global STs were noticed by MLST. The results noted a high prevalence of hvKp strains. Molecular characterization of bacterial strains using NGS/WGS is important to understand the epidemiology of bacterial strains and the antibiotic resistance and virulence genes they are potentially carrying. The data obtained from this study may be utilized to devise careful antibiotic-prescribing approaches and improve patient management practices.

## Introduction

*Klebsiella pneumoniae* (*K*. *pneumoniae*) is one of the pathogenic bacteria listed under the ESKAPE (*Enterococcus faecium*, *Staphylococcus aureus*, *Klebsiella pneumoniae*, *Acinetobacter baumannii*, *Pseudomonas aeruginosa*, and *Enterobacter* species) pathogens [1]. These bacteria utilize mechanisms such as antibiotic resistance and virulence determinants to counteract human defenses and cause invasive diseases. *K*. *pneumoniae *is a versatile gram-negative capsule-forming bacillus associated with several human infections that range from mild to deep-seated invasive infections. Some of the infections frequently associated with *K*. *pneumoniae *include respiratory tract infections (RTIs), urinary tract infections (UTIs), abscesses, and septicemia [2].

Interestingly, *K*. *pneumoniae *can cause community-acquired infections (CAIs) and hospital-acquired infections (HAIs) [3]. CAIs spread among people while moving socially, in places such as classrooms, movie theaters, parks, recreation centers, and other crowded areas. HAIs are contracted by people after 48 hours of hospital admission, wherein they develop an infection different from the condition for which they were hospitalized. Patients admitted to the intensive care units (ICUs) are predisposed to HAIs.

Extended-spectrum β-lactamase (ESBL) and carbapenem-resistant *K*. *pneumoniae *(CR-Kp) strains have been spreading globally and contributing to treatment failures [4,5]. The antimicrobial resistance (AMR) gene *bla*_OXA_ is one of several genes identified in *K*. *pneumoniae *isolates that contribute to carbapenemase activity and resistance to the carbapenem group of antibiotics, which are generally employed as last resort antimicrobial agents to treat infections with multidrug-resistant (MDR) bacteria [6]. Other genes that confer MDR identified among *K*. *pneumoniae *clinical isolates include *bla*_CTX-M_, *bla*_SHV_, *bla*_TEM_, ompK36, and ompK37 [7].

A few strains, characterized as hypervirulent *K*. *pneumoniae *(hvKp), have been associated with severe and invasive infections among healthy and immunocompetent individuals. These strains are known to harbor virulence genes such as *iro *(salmochelin biosynthesis), *iuc *(aerobactin synthesis), and *rmpA *(regulator of mucoid phenotype) [8].

Therefore, it is important to improve our understanding of the sequence types (STs) and serotypes of *K*. *pneumoniae*, the AMR genes, plasmids, and the virulence genes they are potentially carrying.

## Materials and methods

An observational, analytical, and cross-sectional study was conducted among 18 MDR *K*. *pneumoniae *isolates acquired from patients attending Prathima Institute of Medical Sciences, Karimnagar, India. The study period was between April 2018 and April 2020. All isolates were identified, and antimicrobial susceptibility profiles were determined through conventional microbiological techniques and confirmed by automated systems [9-12]. Additionally, all the isolates were investigated using next-generation sequencing (NGS) or whole-genome sequencing (WGS) to identify the genes coding for resistance, such as ESBLs, metallo-beta-lactamases (MBLs), and virulence genes. Multilocus sequence typing (MLST) was carried out to determine the sequence types, and serotyping was carried out to evaluate the capsular polysaccharide (K) and cell wall/lipopolysaccharide (O) serotypes carried by the isolates.

Whole-genome sequencing and genomic characterization for resistance and virulence genes and sequence types

The deoxyribonucleic acid (DNA) was extracted from *K*. *pneumoniae *isolates using the Qiagen QIAamp DNA Mini kit (Qiagen, Hilden, Germany) following the manufacturer’s instructions. Double-stranded DNA libraries with 450 base pairs (bp) insert size were prepared and sequenced on the Illumina platform with 150 bp paired-end chemistry. The genomes that passed sequence quality control were assembled using Spades v3.14 [13] to generate contigs and annotated with Prokka v1.5 [14]. Species identification was carried out using a bactinspector, and contamination was assessed using confindr. All the quality metrics were combined using MultiQC and Qualifyr to generate web-based reports. MLST, AMR, and virulence factors were identified using the ARIBA tool v2.14.4 [15] with BIGSdb-Pasteur MLST database, National Center for Biotechnological Information (NCBI) AMR-acquired gene, PointFinder databases, and VFDB, respectively [16-18]. All the bioinformatic analyses were conducted using Nextflow pipelines developed as a part of the Global Health Research Unit (GHRU), United Kingdom, for AMR surveillance.

Kleborate analysis

The Kleborate tool was used to confirm the species, genes for AMR, and virulence and evaluate the capsular polysaccharide (KL) and cell wall/lipopolysaccharide (O) serotypes [19].

## Results

The mean age of the patients was 46.11±20.35 years. Among the patients included, 12 (66.66%) were males and 6 (33.33%) were females. Among the samples included were blood (2/18; 11.11%), sputum/respiratory secretions (3/18; 16.66%), pus/wound (4/18; 22.22%), and urine (9/18; 50%). A high percentage (>50%) of hvKp strains that had genes coding for AMR and plasmids having the potential to carry *bla*_NDM_ and resistance genes were observed. Of the 18 isolates, 16 (88.88%) revealed the presence of multiple AMR and virulence genes, with evidence of at least one gene coding for beta-lactamase resistance. The study identified the presence of aerobactin (AbST) and yersiniabactin (YbST) STs based on the occurrence of virulence genes such as *iuc *(A-D) and iutA for AbST and *ybt *(A, E, P, Q, S, T, U X, 9, 10, 14, 15, 16), and *irp *(1-2) for YbST. The genes coding for AMR and virulence identified in this study are detailed in Table 1.

**Table 1:** Antibiotic resistance and virulence genes along with their functions *bla*: beta-lactamase gene, MBL: metallo-beta-lactamase, RND: resistance-nodulation-cell division, *bla*_NDM_: New Delhi metallo-beta-lactamase, Ble: bleomycin gene, MDR: multidrug resistance, ATP: adenosine 5'-triphosphate, ABC: ATP-binding cassette, MFS: major facilitator superfamily, RNA: ribonucleic acid, SMR: small multidrug resistance, ESBLs: extended-spectrum beta-lactamases, SDS: sodium dodecyl sulfate

Resistance/virulence genes	Function
*aac *(3IIa, 3IIe, 6Ib, 6Ibcr5)	Aminoglycoside N-acetyltransferase
*aad* (A1, A2)	Ant3 Ia family aminoglycoside nucleotidyl transferase
acrR	Multidrug efflux pump regulator
*aph *(A6, 3Ia, 3Ib, 3VI, 6Ic, 6Id)	Aminoglycoside-o-phosphotransferase
arr2	Integron-encoded rifampin adenosine diphosphate-ribosyl transferase
armA	High-level aminoglycoside resistance
*ompK *(35, 36, 37)	Outer membrane protein of *Klebsiella*
*bla*_AFM-1_	Subclass ‘B1’ MBL
*bla*_CTX-M-15_	Class ‘A’ ESBL
*bla*_NDM-1_, *bla*_NDM-5_	Subclass ‘B1’ MBL
*bla*_OXA_ (1, 9, 181, 232)	Class ‘D’ ESBL oxacillin hydrolyzing
*bla*_SHV_ (11, 26, 27, 28, 67, 89, 187, 212)	Class ‘D’ ESBL carbapenem hydrolyzing
*bla*_TEM_ (1D, 90)	Class ‘A’ broad spectrum ESBL
*ble*-MBL	Bleomycin binding protein
*cat *(A1, A2, B, B3, B4)	Chloramphenicol O-acetyl transferase
*dfr *(A12, A14)	Trimethoprim-resistant dihydrofolate reductase
ereA	Erythromycin esterase
ermB	23SrRNA adenine N methyltransferase
*fos *(A, A6, A7)	Fosfomycin resistance-hydrolyze drugs
ICEKp	Integrative conjugative elements (ICEs) of Klebsiella pneumoniae
*mph *(A, E)	Macrolide 2’ phosphotransferase
msrE	Resistance to erythromycin and streptogramin B
*oqx *(A5, A7, A10, B11, B19, B20)	Low to intermediate resistance to quinoxalines, quinolones tigecycline, nitrofurantoin, and detergents and disinfectants (benzalkonium chloride, triclosan, and SDS)
sat2	Multi-drug efflux RND transporter periplasmic adaptor subunit
qacE delta1	Quaternary ammonium compound efflux SMR transporter
*qnr *(B1, S1)	Qionolone resistance
*rmt *(B, F, F1)	16SrRNA guanine methyl transferase-New aminoglycoside resistance, MDR
*sul *(1, 2, 3)	Sulphonamide-resistant dihydropteroate synthase
*tet *(A, D)	Tetracycline efflux MFS transporter
gyrA	Quinolone resistance
parC	Quinolone resistance
ramR1	Tigecycline resistance
rpoB	Resistance to rifampicin
*str *(A, B)	Aminoglycoside resistance
YbST	Yersiniabactin sequence type
*ybt *(A, E, P, Q, S, T, U X, 9, 10, 14, 15, 16)	Yersiniabactin ABC transporter ATP-binding/permease protein
*irp *(1, 2)	Yersinia gene involved in the synthesis of siderophore yersiniabactin
AbST	Aerobactin sequence type
*iuc *(A-D, 5)	Aerobactin-invasive disease-hypervirulence-hypermucoviscous
iutA	Iron/siderophore acquisition system-ferric aerobactin receptor
infB	Translation initiation factor-engaging cellular restart mechanisms and regulating the maintenance of genome integrity
gapA	Catalyzes the oxidative phosphorylation of glyceraldehyde 3-phosphate-adhesion
fyuA	Yersiniabactin receptor
mdh	Malate dehydrogenase-adaptation
pgi	Catalyzes the reversible isomerization of glucose-6-phosphate-adaptation
phoE	Outer membrane phosphoporin protein E
tonB	Outer membrane protein to transport siderophores and others
wzi	Outer membrane protein involved in capsule attachment to the cell surface

There was a high prevalence of *bla*_SHV_ (17/18; 94.44%) and *bla*_CTX-M-15_ (16/18, 88.88%) antibiotic resistance genes. Other genes identified included *bla*_TEM_ (83.33%; 15/18) and *bla*_OXA_ (14/18; 77.77%). Two (11.11%) strains each showed the presence of *bla*_NDM-1_ and *bla*_NDM-5_ genes. The virulence genes identified were *gapA*, *infB*, *mdh*, *pgi*, *phoE*, *rpoB*, *tonB*, *iuc*, *iut*, and *ybt*. The strain-wise details of the resistance and virulence genes are detailed in Table 2.

**Table 2:** Strain-wise details of antimicrobial resistance and virulence genes KP: *Klebsiella pneumoniae*, M: male, F: female, D: day, UTI: urinary tract infection

Strain	Age/ sex	Source of specimen	Clinical diagnosis	Resistance and virulence genes detected
KP-270	76/M	Blood	Fever	*acrR*, o*mpK36*, o*mpK37*, *ramR1*, *aac6Ib*, *aadA2*, *arr2*, *bla*_CTX M-15_, *bla*_OXA-232_, *bla*_SHV-212_, *bla*_TEM-1_, *catA1*, *catB*, *catB8aac6Ib*, *dfrA12*, *ereA*, *ermB*, *fosA*, *mphA*, *oqxA10*, *oqxB11*, *qacEdelta1*, *qnrS1*, *rmtF1*, *sul1*
KP-271	37/F	Blood	Fever	*acrR*, *ompK36*, *ompK37*, *ramR1*, *aac6Ib*, *aadA2*, *arr2*, *bla*_CTX M-15_, *bla*_OXA-232_,* bla*_SHV-212_, bla_TEM-1_, *catA1*, *catB*, *catB8aac6Ib*, *dfrA12*, *ereA*, *ermB*, *fosA*, *mphA*, *oqxA10*, *oqxB11*, *qacEdelta1*, *qnrS1*, *rmtF1*, *sul1*
KP-272	57/M	Sputum	Diabetic ketoacidosis-Respiratory distress	*acrR*, *ompK36*, *ompK37*, *ramR1*, *aac6Ib*, *aac3IIe*, *aadA2*, *aph3Ib*, *aph6Id*, *arr2*, *bla*_CTX M-15_, *bla*_OXA-1_, *bla*_SHV-28_, *bla*_TEM-1_, *catB3*, *dfrA14*, *fosA6*, *oqxA5*, *oqxB19*, *qacEdelta1*, *qnrB1*, *sul2*, *tetA*
KP-1141	1day/M	Endotracheal secretion	Respiratory distress	*acrR*, *ompK36*, *ramR1*, *gyrA*, *aac6Ib*, *aac6Ibcr5*, *aadA2*, *armA*, *bla*_CTX-M-15_, *bla*_OXA-1_, *bla*_OXA-232_, *bla*_SHV-28_, *bla*_TEM-90_, *catB3*, *dfrA1*, *dfrA12*, *dfrA14*, *ereA*, *fosA6*, *mphE*, *msrE*, *oqxB20*, *qacEdelta1*, *sat2*, *sul1*, *tetD*
KP-1143	53/M	Urine	Cardiovascular disease-UTI	*acrR*, *ompK36*, *ompK37*, *ramR1*, *aadA2*, *aph3Ia*, *aph3VI*, *aph3Ib*, *aph6Ic*, *aph6Id*, *armA*,* bla*_AFM-1_, *bla*_CTX-M-15_, *bla*_NDM-1_, *bla*_SHV-67_, *bla*_TEM-1_, *ble-*MBL, *dfrA12*, *fosA*, *mphA*, *mphE*, *msrE*, *oqxA7*, *oqxB19*, *qacEdelta1*, *qnrB1*, *sul1*, *sul2*
KP-1144	59/M	Urine	Cardiovascular disease-UTI	*acrR*, *ompK36*, *ompK37*, *ramR1*, *gyrA*, *aac6Ib*, *aph3Ib*, *aph6Id*, *arr2*, *bla*_CTX-M-15_, *bla*_OXA-181_, *bla*_SHV-67_, *bla*_TEM-1_, *catA2*, *catB*, *catB8aac6Ib*, *dfrA12*, *ereA*, *fosA*, *mphA*, *oqxA7*, *oqxB19*, *qacEdelta1*, *qnrB1*, *rmtF1*, *sul2*
KP-1145	53/M	Urine	Cardiovascular disease-UTI	*acrR*, *ompK36*, *ompK37*, *ramR1*, *gyrA*, *aac3IIe*, *aac6Ib*, *aac6Ibcr5*, *aph3Ib*, *aph6Id*, *bla*_CTX-M-15_, *bla*_OXA-1_,* bla*_OXA-232_,* bla*_SHV-89_, *bla*_TEM-1_, *catB3*, *dfrA14*, *ereA*, *fosA6*, *fosA7*, *qnrB1*, *sul2*, *tetA*
KP-1216	55/M	Pus	Hepatic abscess	*ybt16*, *ICEKp12*, *gapA*, *infB*, *mdh*, *pgi*, *phoE*, *rpoB*, *tonB*, *ybt *(A, E, P, Q, S, T, U X), *irp *(1-2), *fyuA*, *aac3IIa*, *aadA2*, *aph3Ia*, *strA*, *strB*, *gyrA*, *parC*, *qnrS1*, *mphA*, *catA2*, *catB4*, *sul2*, *tetA*, *dfrA*, *dfrA12*, *OMPK35*, *bla*_CTX-M-15_, *bla*_OXA-1_, *bla*_TEM-1D_, *bla*_SHV-11_
KP-1217	25/F	Urine	UTI	*ybt10*, *ICEKp4*, *gapA*, *infB*, *mdh*, *pgi*, *phoE*, *rpoB*, *tonB*, *ybt *(A, E, P, Q, S, T, U X), *irp *(1-2), *fyuA*, *aac3IIa*, *aac6Ib*, *aadA1*, *aphA6*, *strA*, *strB*, *gyrA*, *parC*, *qnrS1*, *catB4*, *sul3*, *dfrA14*, *bla*_CTX-M-15_, *bla*_OXA-1_, *bla*_OXA-9_, *bla*_TEM-1D_, *bla*_SHV-28_, *bla*_NDM-1_
KP-1219	22/F	Urine	UTI	*gapA*, *infB*, *mdh*, *pgi*, *phoE*, *rpoB*, *tonB*, *bla*_SHV-27_
KP-1220	27/F	Urine	Renal artery stenosis-UTI	*ybt14*, *ICEKp5*, *iuc5*, AbST, *gapA*, *infB*, *mdh*, *pgi*, *phoE*, *rpoB*, *tonB*, *ybt *(A, E, P, Q, S, T, U X), *irp *(1-2), *fyuA*, *iuc *(A-D), *iutA*, *aadA2*, *rmtF*, *gyrA*, *parC*, *emrB*, *mphA*, *catA1*, *arr2*, *sul2*, *dfrA12*, *ompK35*, *ompK36TD*, *bla*_CTX-M-15_, *bla*_OXA-232_, *bla*_TEM-1D_
KP-1221	35/M	Pus	Orthopedic wound	*aadA2*, *aph31A*, *qnrS1*, *mphA*, *sul *(2-3), *tetA*, *dfrA12*, *bla*_CTX-M-15_, *bla*_SHV-26_, *strA*, *strB*, *ybt14*, *ICEKp5*, *gapA*, *infB*, *mdh*, *pgi*, *phoE*, *rpoB*, *tonB*, *ybt *(A, E, P, Q, S, T, U X), irp (1-2), *fyuA*
KP-1222	26/M	Sputum	Type I respiratory failure	*aac3IIa*, *strA*, *strB*, *gyrA*, *qnrB1*, *catB4*, *sul3*, *dfrA14*, *bla*_OXA-1_, *bla*_SHV-187_, *bla*_TEM-1D_, *bla*_CTX-M-15_, *ybt14*, *ICEKp5*, *gapA*, *infB*, *mdh*, *pgi*, *phoE*, *rpoB*, *tonB*, *ybt *(A, E, P, Q, S, T, U X), *irp *(1-2), *fyuA*
KP-1223	45/M	Urine	UTI	*bla*_SHV-11_, *ybt15*, *ICEKp11*, *gapA*, *infB*, *mdh*, *pgi*, *phoE*, *rpoB*, *tonB*, *ybt *(A, E, P, Q, S, T, U X), *irp *(1-2), *fyuA*
KP-1225	75/F	Tissue	Wound	*aadA2*, *rmtB*, *ybt9*, *ICEKp3*, *gapA*, *infB*, *mdh*, *pgi*, *phoE*, *rpoB*, *tonB*, *ybt *(A, E, P, Q, S, T, U X), *irp *(1-2), *fyuA*, *gyrA*, *parC*, *emrB*, *mphA*, *dfrA12*, *bla*_OXA-1_, *bla*_SHV-28_, *bla*_TEM-1D_, *bla*_CTX-M-15_, *bla*_NDM-5_
KP-1226	45/M	Pus	Diabetic foot ulcer	*ybt10*, *ICEKp4*, YbST, *gapA*, *infB*, *mdh*, *pgi*, *phoE*, *rpoB*, *tonB*, *ybt *(A, E, P, Q, S, T, U X), *irp *(1-2), *fyuA*, *rmtF*, *strA*, *strB*, *pmrB*, *gyrA*, *parC*, *qnrB1*, *mphA*, *arr2*, *sul3*, *dfrA14*, *ompK35*, *ompK36TD*, *bla*_OXA-181_, *bla*_TEM-1D_, *bla*_CTX-M-15_, *bla*_SHV-11_
KP-2159	70/M	URINE	UTI	*ybt9*, *ICEKp3*, YbST, *wzi137 gapA*, *infB*, *mdh*, *pgi*, *phoE*, *rpoB*, *tonB*, *ybt *(A, E, P, Q, S, T, U X), *irp *(1-2), *fyuA*, *aadA2*, *rmtB*, *ermB*, *mphA*, *gyrA*, *parC*, *dfrA12*, *ompK35*, *ompK36TD*, *bla*_OXA-1_, *bla*_TEM-1D_, *bla*_NDM-5_, *bla*_CTX-M-15_, *bla*_SHV-28_
KP-2259	70/M	Urine	UTI	*ybt10*, *ICEKp4*, YbST, *gapA*, *infB*, *mdh*, *pgi*, *phoE*, *rpoB*, *tonB*, *ybt *(A, E, P, Q, S, T, U X), *irp *(1-2), *fyuA*, *rmtF*, *strA*, *strB*, *pmrB*, *gyrA*, *parC*, *qnrB1*, *mphA*, *arr2*, *sul3*, *dfrA14*, *ompK35*, *ompK36TD*, *bla*_OXA-181_, *bla*_TEM-1D_, *bla*_CTX-M-15_, *bla*_SHV-11_

The most frequent *K*. *pneumoniae *serotypes found were KL51:O1v2 (3, 16.66%), KL17:O1v1 (3, 16.66%), and KL64:O2v1 (3, 16.66%). The details of serotypes identified in this study are detailed in Table 3.

**Table 3:** Klebsiella pneumoniae serotypes identified in this study KP: *Klebsiella pneumoniae*, KL: capsule type based on Kleborate analysis

Strain	Serotype	
	K (capsular polysaccharide antigen)	O/v (cell wall lipopolysaccharide antigen and its variant)
KP-270	KL51	O1v2
KP-271	KL51	O1v2
KP-272	KL102	O2v2
KP-1141	KL64	O1v1
KP-1143	KL128	O3b
KP-1144	KL64	O2v1
KP-1145	KL52	OL101
KP-1216	KL-64	O2v1
KP-1217	KL-112	O1v1
KP-1219	KL-109	O2v2
KP-1220	KL-51	O1v2
KP-1221	KL-114	O1v1
KP-1222	KL-21	O3b
KP-1223	KL-63	O1v2
KP-1225	KL-17	O1v1
KP-1226	KL-17	O1v1
KP-2159	KL-17	O1v1
KP-2259	KL-64	O2v1

The MLST was performed using selected/functional virulence genes called housekeeping genes. The details of the plasmid replicons and virulence genes and functions along with their usefulness to the bacteria are shown in Table 4.

**Table 4:** Housekeeping genes and plasmid replicons along with their functions *Col*: colicinogenic plasmid, NAD: nicotinamide adenine dinucleotide, DNA: deoxyribonucleic acid, NDM: New Delhi metallo-beta-lactamase

Virulence gene/housekeeping genes	Function	Usefulness
gapA	Catalyzes the oxidative phosphorylation of glyceraldehyde 3-phosphate to 1,3-bisphosphoglycerate (BPG) using the cofactor NAD	Required for optimal adhesion to human epithelial and endothelial cells
infB	Translation initiation factor-engaging cellular restart mechanisms and regulating the maintenance of genome integrity	Cellular growth during nutritional deprivation
Pgi	Catalyzes the reversible isomerization of glucose-6-phosphate to fructose-6-phosphate-important for cellular metabolism	Constantly adapt to many different environmental challenges
mdh	Malate dehydrogenase	Adaptation of bacteria to the environment (aerobic and anaerobic) and cell growth
phoE	Outer membrane phosphoporin protein E	Facilitates efficient diffusion of phosphate and phosphorus-containing compounds across the outer membrane
rpoB	DNA recombination/repair protein	Protects against oxidative damage in host cells
tonB	Outer membrane protein to transport siderophores and others	Carries out heme acquisition, binding of heme, hemoprotein, and hemophore to their respective outer membrane receptors
*IncFIA*, *IncFIB (pQil)*, *IncFII (pKP91)*, *IncFII (pSE11)*, *IncFIB (K)*, *IncFIBpNDM*, *IncFII (K)*, *IncHI1BpNDM*, *IncFII (pKPX1)*, *IncN.1*, *IncR.1*	Incompatibility (Inc) group	Carry drug-resistance genes like NDM-1, NDM-5, and others
*ColKP3*, c*olRNAI*, c*olpVC*, c*olMG828*	Col-like plasmid replicons	Carry drug-resistance genes

The most common MLST-based STs identified in this study included ST-147 (5/18, 27.77%) followed by ST-231 (3/18, 16.66%) and ST-101 (2/18, 11.11%). The details of the MLST types and the plasmid replicons identified in this study are shown in Table 5.

**Table 5:** Strain-wise sequence types and plasmid replicons identified in this study KP: *Klebsiella pneumoniae*, MLST: multilocus sequence typing, ST: sequence type, *Inc*: incompatibility group, *Col*: colicinogenic plasmid

Strain	MLST type	Virulence/housekeeping genes	Plasmid replicons
KP-270	ST-231	*gapA*, *infB*, *mdh*, *pgi*, *phoE*, *rpoB*, *tonB*	*ColKP3*, *IncFIA*, *IncFIB (pQil)*,* IncFII (pKP91)*, *IncFII (pSE11)*
KP-271	ST-231	*gapA*, *infB*, *mdh*, *pgi*, *phoE*, *rpoB*, *tonB*	*ColKP3*, *IncFIA*, *IncFIB*, *IncFII (pSE11)*
KP-272	ST-307	*gapA*, *infB*, *mdh*, *pgi*, *phoE*, *rpoB*, *tonB*	IncFIB (K)
KP-1141	ST-2096	*gapA*, *infB*, *mdh*, *pgi*, *phoE*, *rpoB*, *tonB*	*ColKP3*, *ColRNAI*, *IncFIB (K)*, *IncHI1BpNDM*
KP-1143	ST-147	*gapA*, *infB*, *mdh*, *pgi*, *phoE*, *rpoB*, *tonB*	*ColpVC*, *IncFIB (K)*, *IncFIBpNDM*, *IncFII (K)*, *IncHI1BpNDM*
KP-1144	ST-147	*gapA*, *infB*, *mdh*, *pgi*, *phoE*, *rpoB*, *tonB*	*ColKP3*, *ColMG828*, *IncFII (pKPX1)*, *IncN.1*, *IncR.1*
KP-1145	ST-38	*gapA*, *infB*, *mdh*, *pgi*, *phoE*, *rpoB*, *tonB*	*ColKP3*, *ColMG828*, *IncFIB (K)*, *IncFII (K)*,* IncN.1*, *IncR.1*
KP-1216	ST-147	*gapA*, *infB*, *mdh*, *pgi*, *phoE*, *rpoB*, *tonB*	*ColpVC*, *IncFIB (K)*, *IncFIBpNDM*, *IncFII (K)*, *IncHI1BpNDM*
KP-1217	ST-15	*gapA*, *infB*, *mdh*, *pgi*, *phoE*, *rpoB*, *tonB*	*ColpVC*, *IncFIB (K)*, *IncFIBpNDM*,* IncFII (K)*, *IncHI1BpNDM*
KP-1219	ST-661	*gapA*, *infB*, *mdh*, *pgi*, *phoE*, *rpoB*, *tonB*	*ColKP3*, *ColMG828*, *IncFII (pKPX1)*,* IncN.1*, *IncR.1*
KP-1220	ST-231	*gapA*, *infB*, *mdh*, *pgi*, *phoE*, *rpoB*, *tonB*	*ColKP3*, *IncFIA*, *IncFIB*, *IncFII (pSE11)*
KP-1221	ST-219	*gapA*, *infB*, *mdh*, *pgi*, *phoE*, *rpoB*, *tonB*	*ColKP3*, *IncFIA*, IncFIB (pQil), *IncFII (pKP91)*,* IncFII (pSE11)*
KP-1222	ST-323	*gapA*, *infB*, *mdh*, *pgi*, *phoE*, *rpoB*, *tonB*	*ColKP3*, *ColRNAI*, *IncFIB (K)*, *IncHI1BpNDM*
KP-1223	ST-111	*gapA*, *infB*, *mdh*, *pgi*, *phoE*, *rpoB*, *tonB*	*ColKP3*, *ColRNAI*, *IncFIB (K)*, *IncHI1BpNDM*
KP-1225	ST-101	*gapA*, *infB*, *mdh*, *pgi*, *phoE*, *rpoB*, *tonB*	*ColpVC*, *IncFIB (K)*, *IncFIBpNDM*, *IncFII (K)*, *IncHI1BpNDM*
KP-1226	ST-147	*gapA*, *infB*, *mdh*, *pgi*, *phoE*, *rpoB*, *tonB*	*ColKP3*, *ColMG828*, *IncFII (pKPX1)*,* IncN.1*, *IncR.1*
KP-2159	ST-101	*gapA*, *infB*, *mdh*, *pgi*, *phoE*, *rpoB*, *tonB*	*ColpVC*,* IncFIB (K)*, *IncFIBpNDM*, *IncFII (K)*, *IncHI1BpNDM*
KP-2259	ST-147	*gapA*, *infB*, *mdh*, *pgi*, *phoE*, *rpoB*, tonB	*ColpVC*, *IncFIB (K)*, *IncFIBpNDM*, *IncFII (K)*, *IncHI1BpNDM*

## Discussion

Among many public health problems encountered in the present time is AMR, which is responsible for treatment failures and results in severe morbidity and mortality. There is an increased concern about the emergence and spread of MDR *K*. *pneumoniae *(MDR-Kp) strains carrying genes for AMR, including the genes coding for carbapenemase that confer resistance to carbapenem groups of antibiotics, such as imipenem and meropenem, that are considered as last resort antimicrobial agents [20].

In this study, ST-147 was the most frequent ST with a serotype combination of KL64: O2v1 (three strains), KL128: O3b (one strain), and KL17:O1v1 (one strain). The ST-147, in combination with KL64, was previously identified among *K*. *pneumoniae *isolated from patients admitted to ICUs [21]. A high percentage (>50%) of hvKp strains that had genes coding for AMR and plasmids having the potential to carry *bla*_NDM_ and resistance genes were observed.

Hypervirulent *K*. *pneumoniae *strains

**Figure 1:**
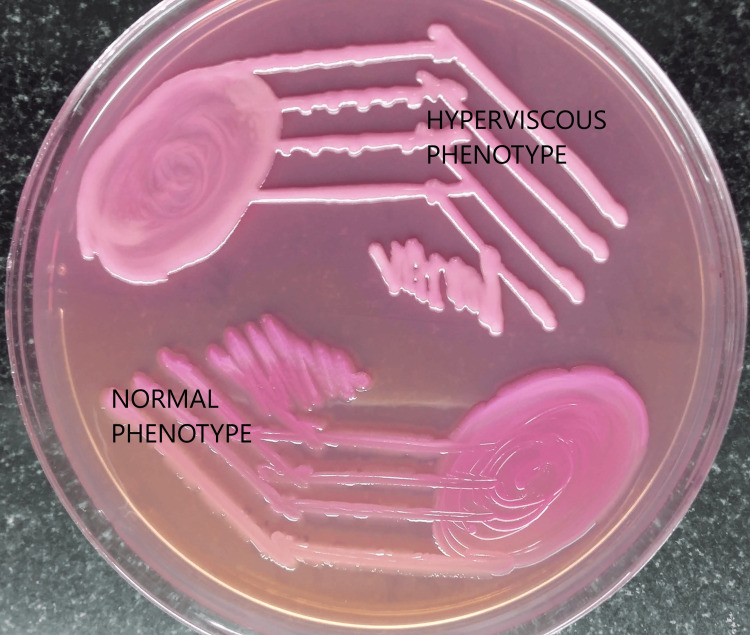
Klebsiella pneumoniae colony textures after being isolated from patient specimens Image credit: Venkataramana Kandi

There are no recommended criteria for classifying *K. pneumoniae *isolates into hypervirulent (hvKp) types. In the clinical setting, the string test can help in the provisional diagnosis of hvKP infection. The test was considered positive if a viscous string measuring more than 5 mm long was obtained by pulling bacterial colonies grown on an agar plate with a bacteriology inoculation wire loop or needle [22]. The *K*. *pneumoniae *strains that show hyperviscosity in the texture of colonies on primary isolation from the clinical specimens are preliminarily identified as hvKp strains (Figure 1).

The hvKp strains are known to secrete increased quantities of capsular polysaccharides due to the *rmpA* and *rmpA2* genes. Further, hvKp strains carry specific plasmids such as pPMK-NDM, which carry AMR genes, and virulence genes such as *iuc*, *iro*,* *and *iut*. *K. pneumoniae *strains having genetic markers for the biosynthesis of salmochelin (*iro*), aerobactin (*iuc*), yersiniabactin (*ybt*), and colibactin (*col*) have been considered as hvKp strains. Besides, the presence of Integrative and conjugative elements of *K*. *pneumoniae *(*ICEKp*) indicates hypervirulence [8]. The present study demonstrated a high percentage (>50%) of hvKp strains, which was considerably high compared to a recent study from Italy that noted a hvKP prevalence of 3.7%. This study observed that hypervirulence was associated with ST-23 and ST-86, and ST-147 carried a *bla*_NDM_ gene [23]. NDM carriage was noticed among ST-147, ST-15, and ST-101 in our study.

Our study noted one (1/18; 5.55%) aerobactin ST (AbST) in one isolate (ST-231), which revealed *iuc *and *iut *genes. Conversely, a study from Morocco showed only 1.5% of isolates belonging to AbST [24]. Our investigation revealed 11 distinct STs, with ST-147, ST-231, and ST-101 being the predominant ones. A study from South India revealed ST-2096 with hypervirulent markers, including AMR genes (*aadA2*, *armA*, *bla*_OXA-1_, *msrE*, *mphE*, *sul1*, and *dfrA14*), plasmids (*IncFIB*), and virulence determinants (*rmpA2*, *iutA*, and *iucABCD*) in *K*. *pneumoniae *isolated from the blood of hospitalized patients [25]. Interestingly, in our study, ST-2096 was isolated from the respiratory secretions of a neonate (1-day-old) that had AMR genes (*acrR*, *OMPK36*, *ramR1*, *gyrA*, *aac6Ib*, *aac6Ibcr5*, *aadA2*, *armA*, *bla*_CTX-M-15_, *bla*_OXA-1_, *bla*_OXA-232_, *bla*_SHV-28_, *bla*_TEM-90_, *catB3*, *dfrA1*, *dfrA12*, *dfrA14*, *ereA*, *fosA6*, *mphE*, *msrE)*, plasmids (*ColKP3*, *ColRNAI*, *IncFIB (K)*, *IncHI1BpNDM*), and virulence genes (*gapA*, infB, *mdh*, *pgi*, *phoE*, *rpoB*, *tonB*).

In a study from Eastern India, the most frequent capsular serotypes identified were K1, K2, K5, K20, K54, and K57. The same study noted a 3.3% prevalence of hvKp strains, with more than 50% of them showing *bla*_OXA-48_ and *bla*_OXA-181_ [26]. In the present study, KL51, KL64, and KL17 capsular serotypes were predominant. Many strains in our study revealed the presence of *bla*_CTXM-15_, *bla*_OXA-232_, *bla*_TEM_, and *bla*_SHV_.

The results from a recent South Indian study that included 30 *K*. *pneumoniae *clinical isolates demonstrated the prevalence of K1, K2, and K5 capsular serotypes, and 26.66% of them were identified as hvKp strains [27]. The ST-86 and ST-23 found in this study were not detected in our study.

In a study from North India, 11.6% of strains were identified as hvKp. This study noted K64 as the most frequent capsular serotype, similar to our results. ST-2096 was the most common ST, along with others such as ST-231 and ST-43, which were also found [28]. Although ST-231 and ST-2096 were detected in our study, ST-43 was not documented.

A study from Iran that analyzed more than 400 strains of *K*. *pneumoniae* revealed an hvKp prevalence of 4% based on the presence of the *rmpA* gene. This study observed the predominance of K1 and K2 capsular types, and 7.8% of strains had *bla*_NDM_ genes [29]. A higher percentage of hvKp in our study may be attributed to the comprehensive evaluation of the isolates using WGS/NGS for plasmid, AMR, and virulence genes.

A recent study from Turkey that investigated virulence and resistance genes among *K*. *pneumoniae *isolates found that 45% of the isolates were hvKp. The study found *bla*_KPC_ and *bla*_OXA_ as predominant resistance genes; none of the isolates had *bla*_NDM_ [30]. In contrast, our study found 11.11% of strains carrying *bla*_NDM_ genes, and none of the isolates revealed *bla*_KPC_.

Study limitations

This study was conducted among limited numbers of *K*. *pneumoniae *isolates acquired from different clinical specimens. The major limitation of this study is it did not compare the genomic evidence with the phenotypic resistance patterns. Also, this study did not evaluate the chromosomal and plasmid origins of the resistance and virulence genes. Based on the clinical condition, the study did not try to establish any relationship between the serotype or ST and the bacterium's virulence.

## Conclusions

The results from the genomic analysis of *K*. *pneumoniae *clinical isolates indicate a very high percentage of them carrying multiple genes conferring AMR and virulence. Multiple AMR genes coding for carbapenemase resistance and ESBLs were identified in the isolates. The study also recognized plasmids carrying AMR and virulence genes in most isolates that can be potentially transmissible between strains and other bacterial species. Besides, more than half of the isolates included in this study were identified as hypervirulent (hvKP) strains. Screening the clinical isolates from hospitalized patients for the presence of AMR genes, virulence genes, and plasmids through NGS/WGS could improve the understanding of the epidemiological characteristics and invasive disease-causing potential of the bacteria prevalent in the hospital environment.
